# Physical Activity, Sedentary Behavior, and Barriers to Exercise in People Living With Dystonia

**DOI:** 10.3389/fneur.2019.01121

**Published:** 2019-10-22

**Authors:** Alana McCambridge, Rebecca M. Meiring, Lynley V. Bradnam

**Affiliations:** ^1^Graduate School of Health, Discipline of Physiotherapy, University of Technology Sydney, Sydney, NSW, Australia; ^2^Department of Exercise Sciences, Faculty of Science, University of Auckland, Auckland, New Zealand; ^3^Faculty of Health and Medical Science, Centre for Brain Research, University of Auckland, Auckland, New Zealand

**Keywords:** dystonia, physical activity, exercise, sedentary behavior, barriers

## Abstract

**Background:** Dystonia is a neurological movement disorder that presents as sustained or intermittent involuntary muscle contractions causing abnormal postures and movements. Knowledge of dystonia is mostly at the impairment level with minimal understanding of activity and participation limitations. Physical activity (PA) is an important aspect of neurological disease management, with wide-ranging benefits for overall health and quality of life. No studies have quantified PA and sedentary behavior (SB), nor explored barriers to being physically active in people with dystonia.

**Methods:** Participants diagnosed with any form of dystonia completed a mixed-methods anonymous online survey on activity behaviors. The International Physical Activity Questionnaire (IPAQ) and Adult Sedentary Behavior Questionnaire (SBQ) assessed self-reported PA and SB. Barriers to exercise engagement were investigated according to the five-factor social-ecological framework and dystonia-specific questions regarding the impact of exercise on symptoms were included.

**Results:** Two-hundred and sixty-three participants consented to the study (mean (SD) age = 55 (13) years, 76% Female). A large proportion of respondents (40%) reported living with cervical dystonia (CD). Overall, the median (IQR) time spent in walking, moderate, and vigorous activity was 60 (0–120), 120 (15–300), and 0 (0–13) min/day, respectively. SB time during weekdays was 285.0 (157.5–465.0) min/day and 345.0 (195.0–502.5) min/day on weekends. Fifty-five percent of participants were dissatisfied with their current level of PA and 75% reported dystonia had decreased their level of PA. Fifty-seven percent found their symptoms were worsened during exercise though the after-effects on symptoms varied. Fatigue, motor symptoms, pain, and poor balance were commonly cited limiting factors. Qualitative and quantitative data indicated difficulties with more vigorous intensity activity. The common barriers to engagement were personal and governmental factors, such as physical impairments, lack of funding and lack of trained exercise professionals.

**Conclusion:** While more than half of respondents indicated they were not satisfied with their current level of PA, and exercise primarily worsened their dystonia symptoms, most participants were meeting the minimum guidelines. Future studies should incorporate robust objective methods of PA and SB measurement and explore the causal mechanisms underpinning exercise-induced aggravation of dystonic symptoms to further enhance life participation of people living with dystonia.

## Introduction

Engaging in physical activity (PA) is vital for ongoing health, reducing risk of cardiovascular and comorbid diseases, and maintaining a high quality of life (1). Exercise (or structured PA) is rapidly becoming a core component for rehabilitation of people living with neurological disorders, with positive impacts on various areas of health, and also disease progression (2–4). Global public health recommendations suggest a person should be physically active and limit their sedentary behavior time. Being sufficiently physically active is defined as achieving a minimum of 150 min of moderate to vigorous intensity activity per week (1), while there are currently no definitive guidelines for sedentary behavior (SB) (5). Lower levels of SB are associated with better health outcomes (6). Despite strong evidence in support of the beneficial effects of PA, most people living with neurological disorders do not meet recommended guidelines and are sedentary (2, 4, 7, 8).

Dystonia is a neurological movement disorder, characterized by involuntary muscle contractions causing abnormal and painful postures, repetitive movements, and sometimes tremor of the affected regions (9). Knowledge of PA and SB in dystonia is limited (2–4, 7, 8). Some studies in people with dystonia report that exercise worsens many motor symptoms, amplifying involuntary contractions, postures and tremor (3, 7). However, there has been little systematic investigation into the amount of PA being achieved by people with dystonia, the impact of dystonia on the ability to exercise, nor conversely of the impact of exercise on dystonic symptoms. Understanding participation in activity behaviors is an important step in discerning areas that health practitioners can intervene to ensure people with dystonia are participating in meaningful life activities, engaging in sufficient PA, and limiting their time spent in SB.

Furthermore, an important component to understanding activity behaviors and encouraging PA in neurological populations is to investigate the impacts of, and barriers to, participation in PA or exercise. Only one study to date has investigated impacts on self-reported PA in people living with dystonia affecting the neck (termed cervical dystonia, CD) using a cross-sectional online survey (10). The study found being employed and having high levels of self-efficacy were major contributors to greater amounts of PA in people with CD (10). However, the study did not determine actual PA levels undertaken by people with CD, nor did the authors address SB. A synthesis of literature further investigated factors affecting PA participation in people living with physical disabilities, to enhance the development of strategies to increase PA (11). The authors identified barriers based on a five-factor social ecological framework; intra- and inter-personal, institutional, community, and policy (11). This framework was used in the current study to generate questions regarding barriers to exercise to enhance the translation of findings about barriers into future intervention strategies.

Investigating activity behavior levels, barriers to PA or exercise, and the impact of PA or exercise on dystonic symptoms will be useful for the development of suitable interventions that promote activity in this population. Therefore, the primary aim of this study was to understand the level of self-reported PA and SB in people with dystonia. The secondary aim was to investigate barriers to exercise and explore the impact of exercise on dystonic motor and non-motor symptoms during and after exercise. The hypotheses were that people living with dystonia would not meet recommended levels of PA, and that exercise would aggravate dystonic symptoms providing a barrier to participation.

## Materials and Methods

Participants diagnosed with any form of dystonia completed a mixed methods anonymous online survey of qualitative and quantitative data on activity behaviors. Ethical approval was provided by the University Ethics Committee (UTS HREC ETH18-3048) prior to survey distribution. Qualtrics online survey software (version XM) was used to deliver the survey to an international audience. Self-reported PA was measured using the International Physical Activity Questionnaire (IPAQ) and time spent in SB was assessed using the sedentary behavior questionnaire (SBQ). The IPAQ asks participants to recall the time spent performing either walking, moderate or vigorous intensity activities in four domains (work/occupational, household, travel, leisure time) for the previous week (12). The SBQ assesses the amount of time spent doing nine sedentary behaviors (watching television, playing computer/video games, sitting while listening to music, sitting and talking on the phone, doing paperwork or office work, sitting and reading, playing a musical instrument, doing arts and crafts, sitting and driving/riding in a car, bus, or train) per day on a typical weekday and weekend (13). Participants were also asked to state whether the activity they reported was usual of their current activity and whether they were satisfied with their current level of PA. Participants completed a series of questions regarding the impact of exercise on their dystonic motor symptoms using closed and open-ended questions, developed in collaboration with a person living with CD (see Supplementary Material 1). Finally, barriers to exercise were investigated using closed-ended questions informed by the social ecological barriers framework (11) and open-ended questions about barriers and enablers. The survey was advertised globally, appearing on Dystonia Support Group webpages in Australia and Sweden, and distributed via email lists, newsletters, and social media in New Zealand, South Africa, the United Kingdom and United States of America. The online survey was available to participants for 4 weeks in 2019.

### Data Analysis

Data were exported into Excel spreadsheets for analysis. Demographic data were analyzed descriptively. The IPAQ and SBQ were scored and analyzed according to established methods (12, 13). StataI/C (version 15.1, StatCorpLLC, TX USA) was used to analyse data. The data from the IPAQ were scored within each activity domain (occupational, household, travel, leisure time related activity) and overall. IPAQ scores were calculated by first summing the time spent in each activity category (walking, moderate or vigorous) within each domain, then multiplying the number of minutes by the number of days of the week the activity was performed during the week. The final step was to multiply by a metabolic equivalent (MET) value (3.3. for walking, between 3.0 and 6.0 for moderate intensity activities and 8.0 for vigorous intensity activities). Therefore, IPAQ scores are reported in MET minutes/week (MET-min/wk). Classifications into activity categories based on recommendations by the WHO were those participants who achieved a minimum of 600 MET-min/wk (considered moderate) and those who achieved 3,000 MET-min/wk (considered high) (14). In addition, for each participant the total amount of time per day spent in walking, moderate, and vigorous activity was summed over all domains and reported as minutes per day (min/day). Data are reported as median values and inter-quartile ranges. The total daily reported time spent in sedentary behavior from the SBQ was calculated separately for weekdays and weekends. Data are represented median values and inter-quartile ranges. Questions regarding the impact of exercise on dystonia symptoms and barriers to exercise were analyzed descriptively for each question by calculating the proportion of responses for each answer in relation to the total number of responses. Inductive analysis determined themes from the open-ended questions and applied where relevant to the social-ecological barrier framework (11).

## Results

### Demographics

Two-hundred and sixty-three participants completed the demographic section. The mean age was 56 years (range 19–83) and 199 identified as female. There were 353 responses to the question regarding dystonia type (participants could select multiple dystonia types). The types of dystonia were broadly grouped, related to the area affected. Focal dystonia on the neck (i.e., CD) was the most common type of dystonia reported with 142 (40%), followed by focal or segmental dystonia involving the hand or foot (*n* = 55, 16%), focal or segmental dystoia involving the face (e.g., craniofacial or oromandibular dystonia; *n* = 44, 12%), blepharospasm (*n* = 39, 11%), generalized dystonia (*n* = 36, 10%), spasmodic dysphonia (*n* = 19, 5%), and “other” (*n* = 18, 5%). As the CD sub-group was relatively large, we have displayed CD data where possible. At the time of the survey, 49% of all respondents had been living with dystonia for more than 10 years. Fifty-three percent were currently undergoing botulinum toxin (BTX) injections for their dystonia, 20% reported they had been treated in the past with BTX but have now discontinued treatment, and 27% had never had BTX injections. One hundred and 63 respondents (62%) were currently taking oral medication to manage dystonic symptoms and of these 149 participants were taking at least one psychoactive drug.

### IPAQ and SBQ

**Figure 1:**
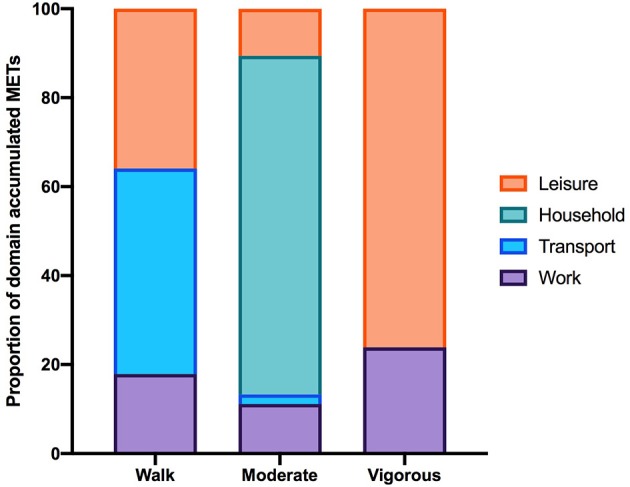
The proportion of the total MET-min/wk in each activity category accumulated per IPAQ domain. Data excludes participants who did not report accumulating activity in any of the activity categories therefore the number of participants in each activity category is 190, 203, and 69. Note that in the household domain activities are all assigned MET values within the moderate-intensity range.

**Figure 2:**
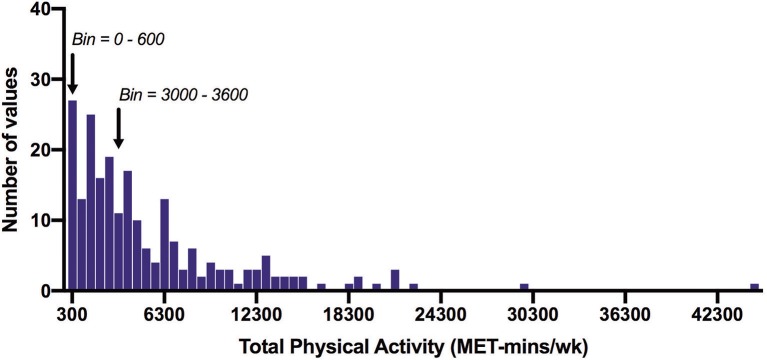
Frequency distribution of total physical activity time measured with the IPAQ (MET-min/wk). Arrows indicate the 600 MET-min/wk and 3000 MET-min/wk thresholds. Of all participants, 12% did not achieve 600 MET-min/wk, and 45% did not achieve 3000 MET-min/wk. Bin width = 600.

Of the total number of respondents, 45 did not report on any PA in the IPAQ and 46 did not report on any SB and were excluded from each analysis. The number of respondents analyzed for the IPAQ and SBQ were 220 and 219, respectively. Of the 220 people who reported activity on the IPAQ, 190 reported accumulating activity by walking, 203 reported accumulating activities of moderate intensity and only 69 reported accumulating activities of vigorous intensity. The participants excluded were those who reported zero activity in any of the walking, moderate or vigorous intensity categories. Figure 1 shows the proportion of accumulated METs of the total METs within each activity category (walking, moderate and vigorous) for each domain (where applicable). To note, 76.2% of the METs accumulated in the vigorous intensity category was done in the leisure time domain though there were only 69 participants that reported any vigorous activity. A large proportion of accumulated METs of moderate intensity (76.1% from 203 respondents) occurred in the household domain, which included garden work and inside housework. The largest proportion of walking METs (46.2% of 190 respondents) were accumulated in the transport domain. For the total physical activity (Figure 2) and per activity category (walking, moderate, vigorous) the data was left-skewed.

Table 1 shows the median (IQR) IPAQ activity scores for all respondents (*n* = 220) within each domain as well as for the total IPAQ score. Overall, the median (IQR) physical activity score was 3,586 (1,617–6,989) (Table 2). Eighty-eight percent of all participants achieved at least 600 MET-min/wk, and 55% achieved 3,000 MET-min/wk.

**Table 1:** IPAQ activity scores for each domain and the total across all domains.

	**Respondents (*n* = 220)**	**CD (*n* = 126)**
**Work**		
Walking	0 (0–17)	0 (0–0)
Moderate	0 (0–0)	0 (0–0)
Vigorous	0 (0–0)	0 (0–0)
Total	0 (0–495)	0 (0–330)
**Transport**		
Walking	256 (0–845)	297 (0–792)
Bicycling	0 (0–0)	0 (0–0)
Total	297 (0–990)	314 (0–792)
**Domestic**		
Inside work	360 (90–1,080)	360 (90–1,080)
Moderate garden/yardwork	240 (0–960)	240 (0–960)
Vigorous garden/yardwork	0 (0–330)	0 (0–165)
Total	1,140 (290–2,940)	1,200 (325–2,940)
**Leisure activities**		
Walking	198 (0–396)	198 (0–396)
Moderate	0 (0–198)	0 (0–88)
Vigorous	0 (0–160)	0 (0–0)
Total	396 (0–1,386)	396 (0–1,320)
**Totals**		
Walking activity	792 (198–2,178)	891 (297–2,376)
Moderate activity	1,680 (479–3,969)	1,647 (478–3,984)
Vigorous activity	0 (0–480)	0 (0–240)
Total physical activity	3,586 (1,617–6,989)	3,281 (1,680–7,110)

*Data are the median (interquartile range) MET-min/wk. Note bicycling and all domestic work are assigned a MET value within the moderate intensity range. CD, cervical dystonia*.

**Table 2:** Total self-reported daily time spent in walking, moderate and vigorous activities and in sedentary behavior across all domains.

**IPAQ**	**Respondents** **(*n* = 220)**	**CD respondents** **(*n* = 139)**
Walking activity	68 (30–140)	75 (30–150)
Moderate activity	180 (60–325)	180 (60–340)
Vigorous activity	0 (0–30)	0 (0–20)
**SBQ**	**Respondents** **(*****n*** **=** **219)**	**CD respondents** **(*****n*** **=** **139)**
Weekday sedentary behavior	375 (225–510)	308 (210–465)
Weekend sedentary behavior	390 (270–525)	360 (240–525)

*Data are median (IQR) minutes/day. IPAQ, International Physical Activity Questionnaire; SBQ, Sedentary Behavior Questionnaire; CD, Cervical Dystonia*.

**Figure 3:**
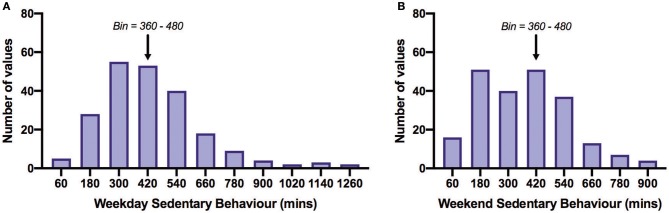
Frequency distribution of daily sedentary behavior time on weekdays **(A)** and weekends **(B)**. All bins above the arrows are those acquiring more than 8 h/day (480 min/day) of sedentary behavior. Of all participants, 27% exceeded 480 min/day on weekdays and 32% on weekends. The overall mean SB time across weekdays and weekends was 401.8 min/day. Bin width = 120.

Table 2 shows PA time in minutes per day spent in each activity category as reported on the IPAQ and SB time as measured by the SBQ. Time spent in SB was 4.75 h/day on weekdays and 5.75–6 h/day on weekends. The distribution of SB time on the weekdays and weekends is shown in Figure 3. The proportion of participants satisfied with their current PA level was 52% and not satisfied 48%. Thirty percent reported that the recalled PA was not typical of their usual activity.

### Dystonia-Specific Questions

Seventy-five percent of respondents indicated dystonia had decreased their amount of PA each week. Participants identified the reasons their dystonia prevented engagement in PA from a provided list of symptoms, of which they could choose multiple reasons (Table 3). The most common symptoms that prevented PA were fatigue, motor impairment, pain and poor balance. During exercise, 57% reported dystonia symptoms were worse and 13% reported symptoms were better (Table 4). After exercise, 34–41% indicated symptoms were worsened and 19–25% reported their symptoms were better (Table 4). Forty-nine percent of respondents reported that their symptoms are affected by exercise immediately or within 5 min of the onset of exercise and 60% of respondents indicated dystonia was aggravated after a single session of exercise (Table 5). Sixty percent of respondents said they could never exercise without symptoms worsening. When asked to elaborate on this, participants said the ability to exercise varied depending on factors such as the type, intensity and amount of exercise, effect of botulinum toxin injections, and how well or unwell they were feeling on a particular day (Table 5).

**Table 3:** Reasons that prevent people with dystonia from engaging in physical activity and exercise.

**Factors**	**Number of responses**	**Percentage (%)**
Fatigue	136	17
Motor symptoms	132	16
Pain	118	15
Balance	95	12
Social embarrassment	68	8
Weak muscles	64	8
Mood	46	6
Vision/Risk of collision with objects/people	45	6
Fear of falling	39	5
Nothing	31	4
Other	27	3
Previous injury	15	2

**Table 4:** Dystonia symptom behavior during and after exercise in the short and long term.

**Factor**	**% Better**	**% Unchanged**	**% Worse**
During exercise	13	30	57
After exercise – same day	25	34	41
After exercise – next day and onwards	19	47	34

**Table 5:** Impact of exercise on dystonia symptoms.

**When symptom change begins**	**Percentage (%)**
Immediately at the onset of physical activity or exercise	25
Soon (5 min) after the onset of physical activity or exercise	24
Symptoms do not change	20
After 30 min of starting physical activity or exercise	14
Unsure	10
More than 1 h after the start of physical activity or exercise	7
**How much exercise is needed to impact on dystonia symptoms**	
A single bout of exercise	60
A period of exercise training (e.g., more than a month of regular exercise)	5
Both	35
**Number of days one could exercise without symptom aggravation**	
Never	61
Every day	16
Three or more times per week	13
Twice a week	6
Once a week	4

Table 6 displays the number of responses for various modes of exercise and the impact on participant symptoms. Most of the exercises were considered to worsen symptoms, however, yoga/pilates and general stretching reduced symptoms for several respondents (Table 6). Vigorous activities like jogging, running, fast cycling and heavy gardening and playing sport negatively affected dystonia symptoms for most respondents. Participants could comment further on this question. Representative participant comments were “*usually anything fast paced or high intensity causes the spasms to be worse*,” “*generally anything that raises the heart rate raises the Dystonia symptoms”* and “*any exercise which results in increased breathing rate causes my spasmodic dysphonia to worsen.”* Participants recommended exercising using a recumbent bike, exercise classes such as yoga. Pilates or tai chi (although some commented that holding poses in yoga caused the tremor and muscle spasms to worsen), classes held in heated room or pool, and gentle stretching and walking were suggested as activities less likely to cause symptom aggravation. Ninety-one respondents reported they had been prescribed exercises for dystonia by a health professional, consisting of gentle stretching and strengthening, posture, balance and mobility exercises and walking. Finally, participants described what happened to their dystonia symptoms during and after exercise. The most common responses included increased muscle spasms, pain, tremor, stiffness and weakness, greater fatigue and reduced balance and coordination and ability to control affected muscles, although many said the response depended on the type of exercise. An example participant comment was “*Increased twisting of head, left shoulder rises, increased spasms, increases strain and tension on muscles on left side of neck and upper back, pain in neck, shoulde r and head,” “the frequency, intensity and strength of the spasms, tremors and involuntary movements all increase,” “my eyes slam shut as soon as I start an exercise class or try to ride my bike.”*

**Table 6:** Different modes of exercise and the impact on dystonia symptoms.

**Factor**	**Number of responses**	**% Better**	**% Unchanged**	**% Worse**
Strengthening/resistance	134	14	30	56
Strengthening without weights	130	21	35	44
Yoga or Pilates	116	39	34	27
General stretching	155	38	37	25
Light walking	176	27	35	38
Brisk walking	153	22	25	53
Jogging	99	11	24	65
Running at a pace that makes it hard to breathe	89	9	20	70
Light cycling	103	12	34	54
Heavy/fast cycling	95	5	26	68
Dancing	95	35	23	42
Aerobics	86	14	30	56
Light gardening	149	12	39	49
Heavy gardening (e.g., digging, cutting trees)	120	7	21	72
Household chores	173	9	36	55
Playing sport (e.g., football, tennis, golf)	84	5	30	65

Symptom behavior during and after exercise responses fell into three categories. First, exercise worsened dystonia during and after exercise, for example “*My neck muscles contract and pull against my head causing significant pain, restricted head movement and often leading to tension headaches that can sometimes last days.”* Second, exercise worsened dystonia during exercise but improved afterwards, for example “*During exercise my tremors, shakes and jerks are much worse. After exercise, they come back to normal levels. Exercise helps keep my fatigue under control.”* Third, dystonia was better during exercise but worse afterwards, for example “*During exercise my spasms ‘feel' less intrusive. But once I stop, the symptoms and pain can be significantly increased.”* The other main message arising from participant responses to this question was that type and intensity of exercise was a factor in symptom aggravation and it was easy to “over do” it and suffer the consequences. Representative participant comments were “*I have to be careful not to overdo it as then the tremors and spasms and pain can become pretty intense,”* and “*This varies greatly, usually they subside but sometimes worsen during exercise. After exercise they are generally better but are much worse if I over-exercise.”* There were a few participants reporting benefits of exercise for their dystonia, such as “*I feel more relaxed and do not feel I am fighting against my neck as much as normal,” “Exercise gives me more energy throughout the day and I feel better for it and proud of my accomplishments,”* and “*My muscles feel really stiff before exercise. As I get warmer the muscles begin to relax and the pain and tremor reduces a lot.”*

### Barriers

The most common barrier to PA was the personal factor “physical and bodily impairments” (41%). The next common personal factor was “negative emotions make it hard to engage in physical activity” (16%). Neither relationship or community level factors were identified as significant barriers to exercise (Supplementary Material 2). The main governmental barriers to exercise were that exercise professionals were not trained in a way that met their needs (18%), the lack of funding for exercise programs (23%), and cost to the individual (18%). The open-ended questions yielded similar results; the most common barriers were intrapersonal, institutional and policy level factors. Participants expressed the desire to return to a wide variety of moderate to vigorous activities, including team sports as well as activities around the house such as gardening and family activities. Participants felt access to appropriate exercise classes or gyms was a barrier due to cost and transportation issues. To further identify barriers to PA, participants were asked “what they thought would help them to become more physically active?” Answers were analyzed according to the five-factor model. The most common answers were intrapersonal; less worsening of symptoms with activity, less fatigue, pain and tremor, better range of motion, more consistent results from botulinum toxin treatment, and increased confidence and motivation. The main interpersonal factor cited was the lack of opportunity to exercise with others. The institutional factor was the lack of exercise professionals who understand dystonia prescribing appropriate exercises and the policy factor was to reduce the cost of participating in exercise programs.

“*Dystonia is an orphan disease. This affects not only research into cause(s) and treatment(s) but also medical and community awareness, knowledge and support. In my experience as a very long term dystonia sufferer there are no meaningful support structures to encourage/assist sufferers to engage in regular exercise, no programs specifically designed to facilitate the process nor medical/exercise professionals trained to provide such support or assistance*.”

## Discussion

The current study was a mixed-methods exploratory investigation of PA and SB in people living with dystonia, their perceived barriers to PA, and impact of exercise on dystonic symptoms. The main findings were that a large proportion of people self-reported engaging in walking and moderate levels of activity, especially in the transport and household domains, respectively. Total physical activity met the minimum recommended guidelines set by the WHO, yet over half of all respondents were dissatisfied with their current level of PA and three-quarters reported dystonia interfered with their ability to be physically active. The most common barrier to PA was the personal factor “physical and bodily impairments,” followed by governmental factors regarding the lack of funding for exercise programs and few trained exercise professionals able to meet their needs. Questions about the effects of exercise on dystonia symptoms revealed a tendency for exercise to worsen symptoms, although some people experience no change or even beneficial effects following exercise. Interestingly, a common theme was that the mode and intensity of exercise was an important factor in symptom aggravation, and that it was easy to “over do” it and suffer the consequences.

The WHO guidelines recommend a minimum of 600 MET-min/wk for health benefits, approximately equivalent to 140–150 min of brisk walking (1). Contrary to our expectations, 88% of all dystonia respondents scored above 600 MET-min/wk suggesting most people with dystonia that were included in our sample are meeting the minimum recommended level of PA for adults. However, findings must be considered with regards to the inherent risks of bias in self-report survey research, such recall bias and over-reporting. In our study walking and moderate intensity activities were the largest contributor to PA time, with minimal contribution from vigorous intensity activities. Walking or low intensity activity was performed during transport related activities, and moderate intensity activity during domestic duties both inside the home and outside garden work. Time spent physically active was not often accumulated during work, although the number of respondents in full-time and/or paid work was not captured in our survey. Leisure time was the greatest contributor to vigorous intensity activity, but the actual time spent at a vigorous level of activity was markedly low, as was the number of participants that performed any vigorous activity. The lack of vigorous intensity exercise may relate to several factors identified in the qualitative analysis. For example, respondents who felt that exercise aggravated their symptoms may be cautious about vigorous intensity exercise as they wished to avoid prolonged exacerbation of symptoms. Some participants also did not feel supported by trained exercise professionals therefore engagement in more vigorous types of activities may have been avoided as they do not feel sufficiently supported or safe to exercise at higher intensities.

Self-reported SB time was considerably lower than that reported in a large-scale study of healthy adults in the US [National Health and Nutrition Examination Survey (NHANES) 2003/4 and 2005/6 (15)]. In the current study, respondents reported spending an overall average of 401.8 min/day (standard deviation in SB while men and women from NHANES spent an average of 490.8 and 484.5 min/day in SB, respectively). To date, there are no widely accepted recommended guidelines for SB however some studies suggest risk of mortality is increased when SB time is more than 8 h/day (16–18). The NHANES study measured PA and SB objectively using accelerometery, whereas the present study used self-reported SB time with the IPAQ. In a study that examined both self-reported and accelerometery-based SB time in people with multiple sclerosis, self-reported SB time was 505.6 min/day while the accelerometer detected SB time was 548.5 min/day (19). No studies to date have reported on self-reported or objectively measured SB in people living with dystonia. As self-reported SB time has been suggested to underestimate accelerometery-based estimates (16), further studies are needed to objectively measure activity behavior in people with dystonia to accurately categorize the amount of SB time and potentially identify areas for improvement.

Our analysis of self-reported PA and SB is the first study to show that a high proportion of people with dystonia are currently meeting public health recommended guidelines. However, there is a growing body of evidence suggesting the current recommendations may be too conservative. In a dose-response meta-analysis of PA, a higher level of PA was strongly associated with a lower risk of five chronic diseases (breast cancer, colon cancer, diabetes, ischemic heart disease, ischemic stroke) (20). For a significant reduction in risk of disease, total PA was shown to be between 3,000 and 4,000 MET-min/wk, several times higher than the minimum 600 MET-min/wk recommendation by the WHO (20). In our data, only 55% of respondents were achieving 3,000 MET-min/wk, indicating that although majority of participants with dystonia are accumulating some time spent physically active, only half are accumulating a dosage that will have meaningful effects on their risk of developing chronic disease. About half of participants were dissatisfied with their current level of PA, a similar proportion to those achieving 3,000 MET-min/wk. Supporting people with dystonia achieve their desired dosage of PA and promoting a dosage that will significantly reduce the risk of cardiovascular disease should be a component in the long-term care of people with dystonia or any chronic neurological movement disorder.

Achieving a higher dosage of PA is possible by increasing the volume and/or intensity of exercise (i.e., structured PA) or incidental PA performed each week. However, for most people in our study exercise exacerbated the symptoms of dystonia, particularly with high intensity activities, and symptoms became aggravated immediately or very soon after the onset of a single bout of exercise. Because of the known relationship between voluntary movement and the presentation of dystonic contractions (9) exercise in this population is inherently difficult, but nonetheless important for overall health and quality of life. A common theme in the qualitative data was that people with dystonia felt concerned about pushing themselves too hard and exacerbating their symptoms further. This may suggest that people with dystonia could be supported to perform low-to-moderate intensity activity for longer or more frequent periods of exercise to achieve a sufficiently high dosage of PA. The modes of exercise identified in our study that either improved or did not change dystonia symptoms for majority (>50%) of respondents were also the types of exercise that were low-to-moderate intensity. These were general stretching, yoga/pilates/tai chi, light walking, dancing, strengthening without weights, and light gardening. In contrast to the modes of exercise that were more likely to worsen symptoms required a more vigorous intensity (see Table 6; heavy gardening, running, fast cycling, etc.). Encouragingly, despite most people with dystonia reporting their symptoms are worsened during exercise, the after-effects of exercise were slightly more positive. For example, a greater proportion of respondents identified that their symptoms were either improved or unchanged following a bout of exercise compared to those that reported their symptoms were worsened after exercise. The qualitative responses confirmed that symptom behavior during and after exercise is considerably varied, with some experiencing relief but most experiencing symptom aggravation. To support the results of our survey, an empirical investigation into exercise intensity and modality should be conducted to better determine how the intensity and mode of activity impacts on dystonia symptoms. As the presentation of dystonia is considerably varied, it will likely be important to do this for each type of dystonia separately. Then it will become important to understand how people with dystonia can exercise without overly exacerbating their symptoms. Future investigations could explore which modes of exercise are less likely to aggravate symptoms, which mode of exercise is best suited to which type of dystonia, and how to maximize the dosage of PA whilst keeping in mind the difficulty experienced with vigorous intensity activities.

The major barriers that inhibited people with dystonia from being physically active were physical impairments, lack of funding, and lack of trained exercise professionals. Fatigue, motor symptoms, and pain were the most common symptoms that prevented people engaging in PA. Without a curative treatment for the physical impairments of dystonia, exercise professionals need to identify methods to help support people with dystonia stay physically active for their general health and well-being. Interestingly, only 91 respondents had previously received an exercise program from a health professional, supporting their assertions that a major barrier to their engagement was a lack of trained professionals able to meet their needs. As clearly indicated by a study participant, there are limited if not nil support structures specifically dedicated for people with dystonia to encourage or assist them to meet their physical activity needs. Education of exercise professionals about dystonia, the various presentations of dystonia, and what is currently known about the beneficial effects of exercise for people with neurological movement disorders would be the first step toward addressing the governmental barriers to PA engagement. Participants suggested they could be more physically active if their symptoms were less aggravated by exercise, they were less fatigued or in pain, had better range of motion, and/or increased confidence or motivation to exercise. Further development of how to appropriately manage non-motor symptoms of dystonia (e.g., pain, fatigue) and negative emotions (e.g., anxiety, depression) within a multi-disciplinary support structure would likely people with dystonia to perform regular exercise, and being supported by trained evidence-based professionals would likely increase confidence and motivation to exercise as well.

In addition, more consistent effects of BTX injections were also suggested by participants as an area of improvement. Participants may be enabled to perform more exercise and/or incidental physically activity if the consistency and effectiveness of BTX injections were improved. Given that BTX is currently the first-line treatment option for focal and segmental dystonia's (e.g., cranial, cervical, hand) (21), understanding the potential role of exercise in conjunction with BTX treatment is also important to ensure people with dystonia are achieving the best outcomes possible. Although the evidence of exercise/physical therapy for dystonia is scarce, a few small randomized controlled trials (RCT) have been performed. In one RCT (*n* = 40 CD), a combined program of exercise, stretching, massage, and BTX treatment was compared to BTX treatment alone (22). The authors found that the effects of BTX were prolonged in the combined program group, and patient reported outcome measures of activities of daily living and pain scored better in the combined program group vs. BTX treatment alone (22). High-quality RCTs are needed to support this preliminary evidence suggesting exercise could be used to augment BTX treatment, and potentially improve the cost-effectiveness of treatment. Reducing the number of BTX treatment sessions needed per year, the volume of BTX needed per treatment, and enhancing the effects on patient outcomes with the addition of a simple, low-cost exercise/physical therapy program could add significant value to the quality of life for many people with dystonia (22). The effects of an exercise program using aerobic and motor control exercises in conjunction with BTX treatment for people with dystonia is not known. Though it would be interesting to determine if exercise could alleviate some of the unwanted side-effects of BTX treatment and decrease the number of people dissatisfied with, and therefore discontinuing, their BTX treatment (23). Furthermore, public dissemination of high-quality evidence is important to ensure people with dystonia are informed about relevant evidence-based treatment options. Dystonia is usually a life-long movement disorder, and evidence-based self-management strategies may be the most viable option in the long-term management of this condition.

## Study Limitations

There are several limitations to consider when interpreting the results. Firstly, although the IPAQ is a widely used instrument to assess PA at a population-level it does require participants to self-report and recall their PA time from the past week which can bias the results. Some studies have suggested the IPAQ overestimates total PA time (24, 25) and public health recommendations may be too easily achieved when assessed using the IPAQ due to the recording of many incidental activities (26). In comparison to the gold-standard doubly-labeled water method the IPAQ was found to underestimate higher intensity activity (27). The IPAQ is yet to be psychometrically assessed in chronic neurological populations such as dystonia, and administration of an online version of the IPAQ needs to be validated also. The administration of the IPAQ online without supervision could have contributed to the number of participants that scored the IPAQ inappropriately in our study. Forty-five participants reported a total PA time of 0 min/wk which is not possible if the IPAQ is answered correctly and the cases were excluded from analysis. Quantifying PA and SB time in people with dystonia using objective methods of assessment, such as accelerometery or doubly labeled water are needed. In addition, as the survey was only available online people without access to the internet could not participate. Furthermore, the online survey was available to people from all countries so cross-cultural differences may have also influenced the results. In relation to the qualitative results and barriers to exercise, some closed-ended questions may have limited the ability of participants to accurately record their lived experience. Future qualitative studies could utilize focus groups or interviews to obtain more in-depth data about the perspectives of people living with dystonia and the barriers they experience. Follow-up studies to support the current findings are recommended.

## Conclusions

Knowledge of activity and participation limitations in people living with dystonia is limited. Understanding the factors that impact PA engagement is fundamental to supporting people with dystonia maintain their activities of daily living and meaningful participation in life after diagnosis. Overall, from this self-reported activity data it appears people living with dystonia are achieving the minimum requirements of PA time according to their self-reported activity levels, by means of incidental activity during transport and domestic duties. Future studies should incorporate objective methods of PA and SB measurement such as accelerometery to confirm the results of this study. The immediate and short-term effects of exercise seem to aggravate dystonia symptoms, however the longer-term impact of an appropriately prescribed exercise program with consideration given to the mode and intensity of exercise is not known. Addressing the barriers to PA in this population could lead to meaningful improvements to the well-being and overall quality of life of people living with dystonia.

## Data Availability Statement

The datasets generated for this study are available on request to the corresponding author.

## Ethics Statement

The studies involving human participants were reviewed and approved by University of Technology Sydney (UTS) HREC ETH18-3048. The patients/participants provided their written informed consent to participate in this study.

## Author Contributions

LB: conception of the initial idea. AM, RM, and LB: survey development, survey dissemination, and manuscript preparation. AM: ethical approval and set up online survey. RM and LB: data analysis.

### Conflict of Interest

The authors declare that the research was conducted in the absence of any commercial or financial relationships that could be construed as a potential conflict of interest.
