# Supplementary material for: Physical Activity, Sedentary Behavior, and Barriers to Exercise in People Living With Dystonia
Source: Front Neurol. 2019 Oct 22;10:1121. doi: 10.3389/fneur.2019.01121 (PMC6817622; doi:10.3389/fneur.2019.01121)
Supplement: Supplementary file 1 [file Table_2.DOCX]

Supplementary Material

**1 Supplementary Dystonia-specific Questions**

1. What is it about your dystonia that prevents or limits you from engaging in physical activity? (You may tick more than one option)

- Pain
- Motor symptoms (e.g. muscle spasm or tremor worsens)
- Vision/Risk of collision with objects/people
- Balance
- Fatigue/tiredness
- Mood
- Social embarrassment
- Fear of injury
- Existing injury from dystonia
- Weak muscles
- Nothing about my dystonia prevents me from engaging in
- physical activity
- Other (please describe):

1. To what extent has dystonia affected the amount of physical activity you do per week?

- Greatly decreased
- Slightly decreased
- No change
- Slightly increased
- Greatly increased

1. What happens to your dystonia symptoms during physical activity or exercise? This may include any kind of physical activity such as walking, household chores, or exercising at a gym.

- Symptoms get worse during exercise
- No change to my symptoms
- Symptoms get better during exercise
- I never perform any kind of physical activity or exercise

1. If your symptoms are changed by exercise, when do you notice they change? (You may select more than one option if needed)

- Immediately at the onset of physical activity or exercise
- Soon (5 mins) after the onset of physical activity or exercise
- After 30 minutes of starting physical activity or exercise
- More than 1 hour after the start of physical activity or exercise
- I am unsure
- Symptoms do not change

1. What happens to your dystonia symptoms once you have stopped performing physical activity or exercise in the short term (i.e. on the same day)?

- Symptoms are worse
- Symptoms are slightly worse
- Symptoms are the same
- Symptoms are slightly better
- Symptoms are better

1. What happens to your dystonia symptoms once you have stopped performing physical activity or exercise in the long term (i.e. the next day and onwards)?

- Symptoms are worse
- Symptoms are slightly worse
- Symptoms are the same
- Symptoms are slightly better
- Symptoms are better

1. If your dystonia symptoms are changed by physical activity or exercise, does this happen after a single or multiple sessions?

- After a single bout of exercise
- After a period of exercise training (e.g., more than a
- month of regular exercise)
- Both

1. Is there a certain number of days in which you can do physical activity or exercise without your symptoms getting worse?

- Once a week
- Twice a week
- Three or more times per week
- Every day
- Never

1. Please feel free to elaborate on your answer above.
2. Is there a particular type of physical activity or exercise you do that impacts on your dystonia symptoms (i.e, makes them worse or better)? Please tick all types of physical activities that apply to you. You may leave blank those activities that you do not regularly perform.

- Strengthening/resistance (e.g. lifting weights)
- Strengthening without weights (e.g. own body weight squats, lunges, push ups)
- General stretching (e.g. touching toes, stretching side)
- Yoga or pilates
- Light walking
- Brisk walking
- Jogging
- Running at a pace that makes it hard to breathe
- Light cycling
- Heavy/fast cycling
- Dancing
- Aerobics
- Light gardening
- Heavy gardening (e.g., digging, cutting trees)
- Household chores
- Playing sport (e.g., football, tennis, golf)
- Other (please specify):

1. Please feel free to elaborate on your answers above. For example is there a particular type of equipment that you use, do you prefer to exercise indoors vs outdoors, any specific type of yoga, do you exercise alone or with supervision.
2. After your diagnosis of dystonia, have you had any exercises prescribed to you? If yes, please describe who prescribed them to you and what type of exercises they were (e.g., stretching, resistance, cardio).
3. Can you describe what happens to your dystonia symptoms during and after you exercise?
4. In what physical activities would you like to participate, but don’t feel capable of performing for any reason?
5. In what physical activities would you like to participate, but don’t have access to for any reason?
6. What do you think would help you to be more physically active?

**2 Supplementary data on Barriers to Exercise**

| **Personal factors** | **Number of respondents** | **% of responses** |
| --- | --- | --- |
| **Physical and bodily impairments from dystonia make it hard for you to exercise or engage in physical activity** | 152 | 41.30 |
| **Negative emotions (e.g. anxiety, depression) make it hard for you to exercise or engage in physical activity** | 60 | 16.30 |
| **Other personal barriers not listed** | 37 | 10.05 |
| **None are applicable to me** | 35 | 9.51 |
| **Self-perceptions regarding your ability to exercise or engage in physical activity** | 35 | 9.51 |
| **Employment status (e.g. not enough time in the day due to work commitments) is a barrier** | 31 | 8.42 |
| **Attitudes/beliefs/perceived benefits about exercise or the effects of exercise or physical activity on your dystonia or your health in general** | 18 | 4.89 |

| **Relationship factors** | **Number of respondents** | **% of responses** |
| --- | --- | --- |
| **None are applicable to me** | 131 | 37.86 |
| **Other relationship barriers not listed** | 66 | 19.08 |
| **Social relationships are not available to support you in participating in physical activity or exercise (e.g. lack of other people to exercise with)** | 42 | 12.14 |
| **Lack of social support to encourage you to exercise or engage in physical activity e.g. no interest from others** | 42 | 12.14 |
| **Lack of friend or family support** | 25 | 7.23 |
| **Lack of acquaintances, peers, colleagues, neighbours and community members support,** | 24 | 6.94 |
| **Attitudes of others in society towards you participating in physical activity or exercise** | 16 | 4.62 |

| **Community level factors** | **Number of respondents** | **% of responses** |
| --- | --- | --- |
| **None are applicable to me** | 134 | 38.73 |
| **There is a lack of information regarding what’s on offer in the community for exercise and physical activity** | 53 | 15.32 |
| **A lack of a cohesive relationship between groups and organizations offering healthcare and physical activity or exercise in your local community makes it hard to understand how to participate in exercise or physical activity or what to actually do** | 32 | 9.25 |
| **The local climate does not encourage you to exercise or engage in physical activity** | 21 | 6.07 |
| **Other community factors not listed** | 14 | 5.36 |
| **There is nowhere suitable for you to exercise (e.g. local park, swimming pool, gym) in your local area** | 7 | 2.02 |

| **Policies/Governmental level factors** | **Number of respondents** | **% of responses** |
| --- | --- | --- |
| **None are applicable to me** | 88 | 27.85 |
| **There is no funding for exercise and physical activity programs for people with dystonia,** | 73 | 23.10 |
| **Staff/professionals within the organization are not trained in a way that meets your needs** | 57 | 18.04 |
| **The cost (money) is a barrier to your participation in exercise or physical activity,** | 56 | 17.72 |
| **Transportation services and/or systems make it difficult for you to participate in exercise or physical activity programs** | 31 | 9.81 |
| **Other (e.g. restrictive policies and bureaucracy at local or national government level): Please explain** | 11 | 3.48 |
